# Cross-link Between CircRNAs and Neuroinflammation in Parkinson’s Disease

**DOI:** 10.1007/s12035-026-06085-3

**Published:** 2026-08-06

**Authors:** Nahla E. El-Ashmawy, Eman G. Khedr, Renad T. Darwish, Amera O. Ibrahim

**Affiliations:** 1 Department of Biochemistry, Faculty of Pharmacy, Tanta University, El-Geish Street, Tanta, 31527 Egypt; 2 Department of Pharmacology and Biochemistry, Faculty of Pharmacy, The British University in Egypt, El Sherouk City, Cairo, 11837 Egypt

**Keywords:** Non-coding RNA, CircRNA, MiRNA, Parkinson’s disease, Neuroinflammation, Astrocytes/microglia activation

## Abstract

Parkinson’s disease (PD) is a major neurodegenerative disorder affecting a large number of people worldwide. PD has been characterized by motor abnormalities, as well as non-motor abnormalities that lower patients’ quality of life. The pathological features of PD include the substantia nigra’s dopaminergic neurons degradation, leading to a progressive clinical course, Lewy bodies and Lewy neurites, which are primarily composed of α-synuclein, and chronic neuroinflammatory changes that contribute to disease progression. Circular RNAs (circRNAs) are a type of circular single-stranded RNAs possessing high stability. Their expression varies depending on tissue type, cell type, and developmental stage, suggesting their roles in regulating biological processes. Recent research has indicated that circRNAs participate in PD pathophysiology by modulating neuroinflammation, immune response, mitochondrial dysfunction, and reactive oxygen species accumulation. Mechanistically, many circRNAs appear to act as molecular sponges for microRNAs, thereby influencing the expression of key genes involved in inflammatory signaling, synaptic regulation, and neuronal survival. This review summarizes the impact of circRNAs on neuroinflammation, astrocyte/microglia dysfunction, mitochondrial damage, and oxidative stress in PD. It also summarizes experimental evidence from cellular and animal models showing that multiple circRNAs can modulate inflammatory pathways in PD and related neurological disorders. However, only a limited number of studies have evaluated circRNAs as biomarkers or therapeutic targets in patient samples, and comprehensive in vivo validation of circRNA-miRNA-target network remains insufficient. A better understanding of these regulatory pathways may help identify clinically relevant biomarkers and support the development of circRNA-based therapeutic strategies for PD.

## Introduction

Parkinson’s disease (PD) has been considered one of the common neurodegenerative disorders, affecting both motor and non-motor health worldwide, resulting in significant disability [1]. More than 1% of people over 65 have PD, which is expected to double in incidence by 2030. Its non-motor symptoms include constipation, anxiety, and cognitive impairment, while its motor symptoms include slow movement (bradykinesia), rigidity of the muscles, and tremor. It has been characterized by the degradation of dopaminergic neurons in the brain’s substantia nigra (SN) [2].

It is significant to note that between 50 and 80% of all nigral dopaminergic neurons become degenerated when patients exhibit motor symptoms. This degeneration can decrease dopamine release in the striatum, thereby changing the pathways that the basal ganglia use to regulate movement. Thus, the activation of basal ganglia nuclei and dopamine replenishment/agonism are currently used to treat PD motor symptoms. Notably, these treatments neither focus on non-motor symptoms nor have an effect on the disease development [3].

The complex etiology of PD results from the interplay of aging, environmental factors, and genetic factors. Although genetic factors have yielded important knowledge into the pathogenesis, they are considered a small percentage of cases; the majority are most likely the result of epigenetic changes brought on by aging and environmental risk factors. Lewy bodies and Lewy neurites have been considered as the most pathological features of PD. They have been shown to consist primarily of aggregated αsynuclein (α-syn) [4]. Neuroinflammation, oxidative stress, mitochondrial dysfunction, and impaired protein clearance due to the defect of autophagy, lysosomal, and ubiquitin-proteasome systems are additional molecular pathogenic pathways [5].

Neuroinflammation and immune response are important factors in PD. Research on animal models of PD has demonstrated that inflammation has an important role in the progression of the disease, making chronic inflammation one of the pathological features of the PD [6]. During neuroinflammatory reactions, activated microglia have been shown to produce a large number of pro-inflammatory cytokines and reactive oxygen species (ROS), which subsequently trigger neurotoxicity. Overexpression of pro-inflammatory mediators, activated microglia, and activated astrocytes have been observed in the SN of PD patients [7].

Autopsies of the brain of PD patients after death have demonstrated the involvement of both innate and adaptive immunity. In PD patients, the pro-inflammatory mediators have been elevated, and the SN and other impacted regions have been observed to contain T-lymphocytes, activated microglia, and astrocytes [6]. Innate and adaptive immunity have also been implicated in the pathophysiology of PD. Innate immunity recognizes common cell signaling pathways, known as pathogen-associated molecular patterns (PAMPs), through toll-like receptors (TLRs), which have been expressed by various cell types, such as neurons, microglial cells, astrocytes, and oligodendrocytes [8]. The aggregation of misfolded α-syn has been reported to activate peripheral monocytes and microglia via TLR4. This triggers to the release of many pro-inflammatory cytokines, such as interleukin 1β (IL-1β) and tumor necrosis factor α (TNF-α), thereby initiating innate immune responses [9].

In addition to innate immunity, adaptive immune mechanisms have been demonstrated to be crucial in PD. The infiltration of lymphocytes across the brain of Parkinson’s patients has been identified as a major component of adaptive immunity. Adaptive immunity may contribute to neuronal damage when CD4+ and CD8+ T-cells enter the brain and react to α-syn peptides. Furthermore, autoantibodies against α-syn have been detected in early PD, but have gradually declined, indicating an active humoral immune response [10].

Circular RNAs (circRNAs) have been identified as a class of single-stranded regulatory RNAs that form circular structures through covalent binding by back splicing and thus lack a 3′ poly A tail and a 5′ end cap. The absence of free ends has distinguished them from conventional linear RNA. CircRNAs have been shown to exhibit a half-life of over 48 h, rather than about 6 h for linear transcripts. Recent research has demonstrated that circRNA expression varies according to tissue, cell, and developmental stage, indicating that they regulate various biological processes [11, 12].

Current research has suggested that circRNAs may be important for the occurrence and progression of multiple acute neurodegenerative diseases, primarily cerebral ischemia [13], epilepsy [14], and traumatic brain injury [15], in addition to chronic neurodegenerative illnesses, such as PD [16], Alzheimer’s disease (AD) [17], and amyotrophic lateral sclerosis [18]. While circRNAs have drawn more attention, in some cases, their biological roles and mechanisms have remained unclear [19].

This review aims to provide an overview of the pathogenesis and neuroinflammatory mechanisms of PD. Furthermore, it highlights the regulatory mechanisms of circRNAs in these processes, focusing on inflammation, astrocyte dysfunction, microglial dysfunction, mitochondrial damage, and oxidative stress.

## Pathogenesis and Neuroinflammatory Mechanisms of Parkinson’s Disease

Numerous processes are believed to be involved in the pathogenic pathway of PD, including the misfolding and aggregation of α-syn, mitochondrial dysfunction, oxidative stress, and neuroinflammation [20].

### α-Synuclein Structure, Aggregation, and Function in Neurotransmission

The α-syn protein has been identified as a soluble protein mostly expressed in the central nervous system’s (CNS) perinuclear and presynaptic terminals, where it has a crucial role in synaptic function. [21]. It is encoded by the SNCA gene and consists of 140 amino acids with a molecular weight of approximately 19 kilodaltons [20]. The structure of α-syn is composed of three domains. The N-terminal amphipathic domain (1–60 Aa) has been shown to mediate α-syn nucleoprotein interaction with the lipid membrane. The central hydrophobic core region, which contains the non-Aβ-amyloid component (NAC) domain (61–95 Aa), has been considered the first location for aggregation of α-syn protein into amyloid fibrils [22]. The C-terminal acidic domain (96–140 Aa), enriched in acidic amino acids, has been reported to participate in nuclear localization and interactions with other proteins, metals, and small molecules [23].

Under physiological conditions, α-syn has been observed to exist mostly in the cytosol as a soluble monomer or to turn into an alpha-helical structure when it binds to the synaptic vesicle membrane [24].

It has been reported that two sequential motifs, P1 and P2, located in the N-terminal region of α-syn are the “main regulators” in the aggregation of α-syn, as these regions are crucial for the transition of the oligomer to fibrils [25]. The native compact conformation of α-syn, resulting from interactions between the NAC domain and the C-terminus as well as between the N-terminal regions, must be maintained to prevent aggregation. The disruption of native compactness, driven by environmental factors (heavy metals, pesticides, toxic chemicals), alterations in SNCA gene expression caused by gene triplication, duplication, or SNCA gene mutations, and/or post-translational modifications (phosphorylation, ubiquitination, and acetylation) can cause α-syn misfolding and aggregation [20, 26]. Conditions such as decreased pH and elevated temperature have been reported to cause partly folded proteins that are susceptible to self-aggregation [27]. The mildly acidic PH values have been found in intracellular sites, such as endosomes and lysosomes [20].

**Fig. 1:**
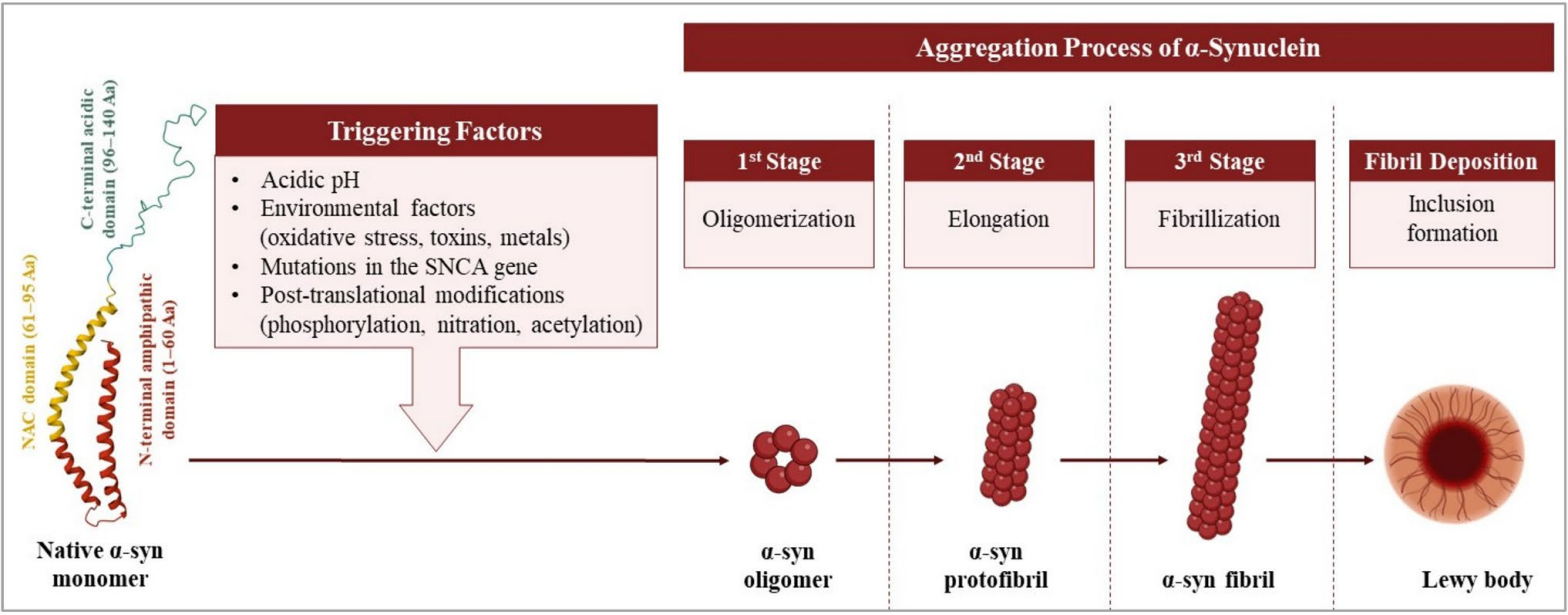
Schematic representation of the structure and aggregation of α-synuclein in Parkinson’s disease. The aggregation process of α-syn occurs primarily in the cytoplasm and has been divided into three stages. The 1 st stage is the rate-limiting step, in which native α-syn monomers oligomerize to form oligomeric species. This stage is enhanced by acidic pH and environmental factors (such as high exposure to heavy metals, air pollutants, pesticides, toxic chemicals, and methamphetamine), mutations in the SNCA gene, and post-translational modifications (such as phosphorylation, ubiquitination, and acetylation). The 2nd stage, called the elongation stage, is when oligomers transform into organized aggregates with extensive β-structure. These aggregates can subsequently develop into fibrillar aggregates. The 3rd stage, the secondary nucleation process, is when the surfaces of pre-existing fibrillar aggregates promote the clumping of monomeric protein units, leading to rapid proliferation of fibers that deposit as insoluble inclusions called Lewy bodies in PD. The structure of micelle-bound human α-synuclein is downloaded from the RCSB PDB database (PDB ID: 1XQ8). NAC, non-Aβ-amyloid component; SNCA, synuclein alpha

The aggregation process occurs primarily in the cytoplasm and has been divided into three stages (Fig. 1). The first stage, the primary nucleation process, has been described as the rate-limiting step in which proteins self-assemble from their native monomeric form to form soluble, amorphous oligomeric species [20]. The second stage, the elongation phase, has been marked by rapid fibril growth, as oligomers can transform into organized assemblies with extensive β-structure, which can then expand into fibrillar aggregates. The third stage, the secondary nucleation process, in which the surfaces of pre-existing fibrillar aggregates frequently promote the creation of new oligomers by causing protein monomers to clump together on the fiber surface, ultimately forming new oligomers. This autocatalytic process can produce rapid fibril proliferation that deposits as insoluble inclusions called Lewy bodies in PD [28].

**Fig. 2:**
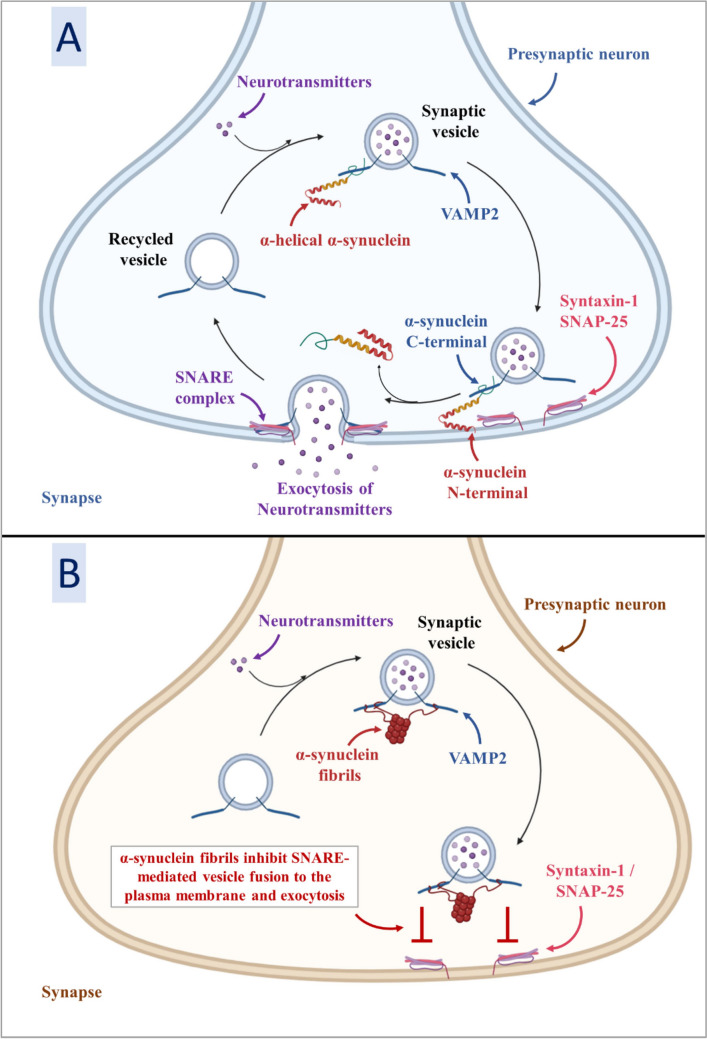
Roles of α-synuclein in vesicle trafficking in Parkinson’s disease under physiological and pathological condition. **A** Physiological condition: α-syn monomers facilitate exocytosis by interacting with the key synaptic vesicle proteins including, VAMP2, syntaxin-1, and SNAP-25. This interaction promotes the formation of the SNARE complex, thereby controlling the exocytosis process. **B** Pathological condition: the accumulation of α-syn aggregates, especially oligomers and preformed fibrils, interferes with presynaptic function through inhibiting the fusion of SNARE-mediated vesicle to the plasma membrane. This inhibition leads to abnormal control of neurotransmitter exocytosis and transmembrane trafficking. VAMP2, vesicle-associated membrane protein 2; SNARE, N-ethylmaleimide-sensitive factor attachment protein receptor

Studies have proposed that α-syn helps in recruiting synaptic vesicles to the plasma membrane using its unstructured C-terminal and amphipathic N-terminal regions. The C-terminal region binds to vesicle-associated membrane protein 2 (VAMP2) on the synaptic vesicle membrane, while the N-terminal region interacts with the plasma membrane of the synaptic vesicle, thereby facilitating exocytosis (Fig. 2). Furthermore, the molecular interaction between α-syn on the synaptic vesicle surface and VAMP2 on adjacent synaptic vesicles has been reported to maintain physiologic synaptic vesicle clustering and promote soluble N-ethylmaleimide-sensitive factor attachment protein receptor (SNARE) complex assembly, thereby regulating synaptic neurotransmitter release [29, 30 ].

The synaptic SNARE complex consists of the VAMP2 on the synaptic vesicle and syntaxin-1 and SNAP-25 on the presynaptic plasma membrane and has been recognized as mediating synaptic vesicle fusion. It has been thought that the membrane-bound form of α-syn on synaptic vesicles functions as a chaperone for the SNARE complex in the presynaptic terminal, helping maintain normal neurotransmitter release and delaying toxicity and disease progression. In contrast, the cytosolic unfolded form of α-syn has been suggested to lack these chaperone capabilities and be more susceptible to aggregation. Under pathogenic conditions, α-syn aggregates have been shown to disrupt the presynaptic function and inhibit the SNARE-mediated vesicle fusion to the plasma membrane, thereby resulting in aberrant transmembrane trafficking and impaired neurotransmitter release [29, 31].

### Neuroinflammation in Parkinson’s Disease

The inflammatory mechanisms have been highly regulated to protect the host from the aggravating agents and encourage tissue healing. Chronic inflammation has been recognized as a defining feature of the pathophysiology of PD [32]. Microglia and astrocytes, two glial cell types found in the CNS, have been identified as important players in the etiology and development of PD. Under physiological conditions, they can continuously monitor the brain parenchyma to preserve CNS homeostasis through the release of neurotrophic factors, the elimination of synaptic glutamate, the reconfiguration and reshaping of synapses, and other processes [33].

Damage-associated molecular patterns (DAMPs) and PAMPs released from disrupted neurons or protein aggregates can activate glial cells, leading to chronic neuroinflammation in PD. A significant pathogenic protein, aggregated α-syn, can be widely detected in neuronal cells throughout various parts of the brain and is also present in biological fluids, such as plasma or cerebrospinal fluid (CSF) [7]. It has been shown that α-syn accumulation cause immune activation, leading to the release of ROS and pro-inflammatory cytokines, which result in the degradation of dopaminergic neurons. Chronic neuroinflammation has appeared to act as a cofactor for the progression of the disease, even though it might not be the causative mechanism in all cases of PD. Inflammation has been strongly linked to genetic susceptibility, traumatic injuries, bacterial or viral infections, environmental pollutants, heavy metals, and pesticides [34].

#### Role of Microglia and Astrocyte in Neuroinflammation

Microglia are considered the primary defense of the innate immune system in the CNS, acting as resident macrophages, with homeostatic roles [35]. In a state of basal homeostatic activity, microglia have exhibited a resting state of activity, where they search the surroundings for indications of infection and cellular distress and react to immunomodulatory chemicals produced by healthy neurons, such as CX3CL1 (fractalkine), cluster of differentiation 200 (CD200), CD22, CD45, CD95, or neural cell adhesion molecule [36]. Misfolded or aggregated proteins like α-syn, DAMPs generated by dying neurons, pro-inflammatory mediators released by astrocytes, bacterial and viral compounds, and signals supplied by TLRs can all activate microglia [32].

**Fig. 3:**
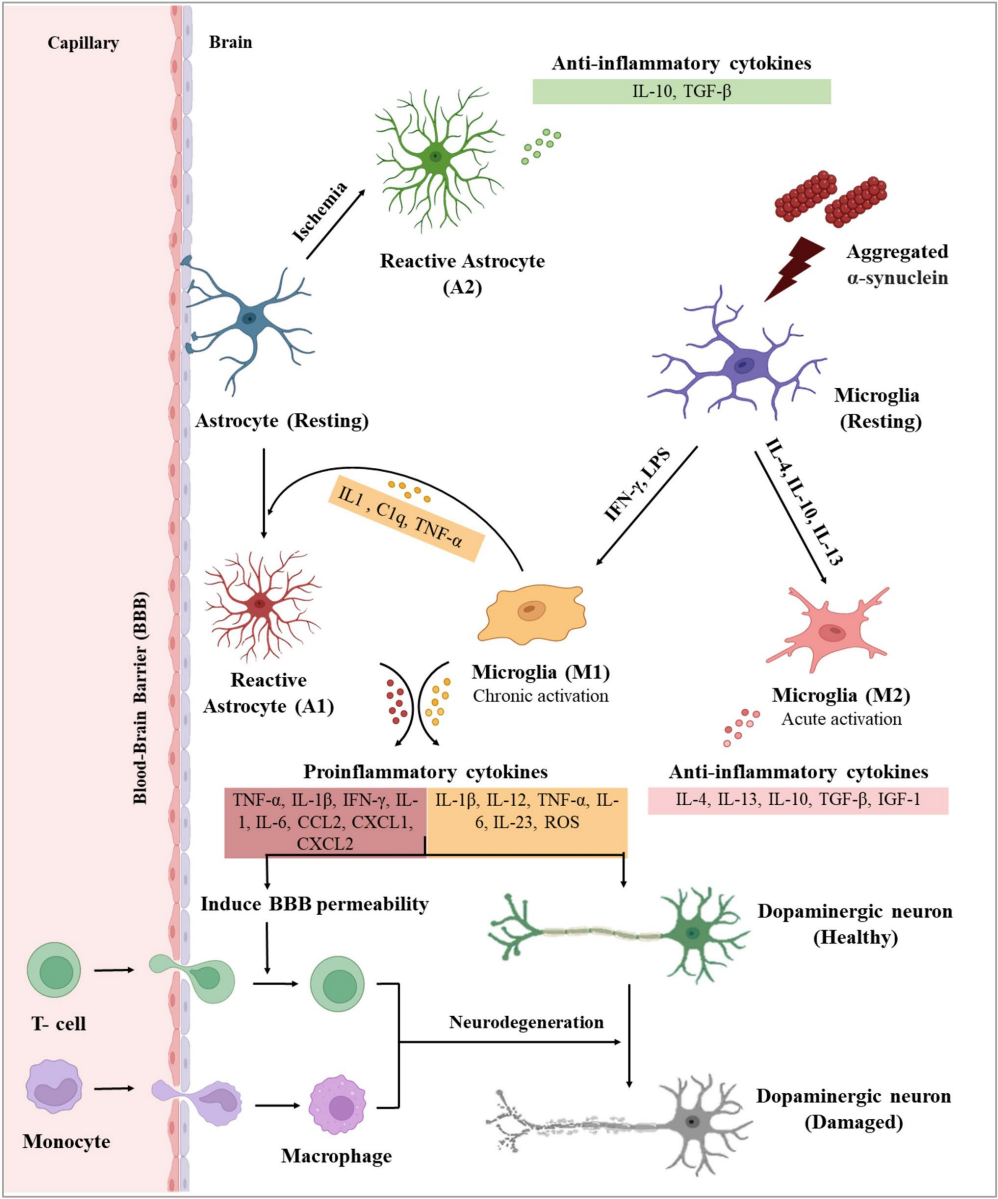
Schematic illustration of the neuroinflammatory cascade in Parkinson’s disease. Aggregated α-syn can activate microglia into pro-inflammatory M1 phenotype (chronic activation) and anti-inflammatory M2 phenotype (acute activation), whereas ischemia can activate astrocytes into neuroprotective A2 phenotype, and inflammatory secretion from M1 microglia can activate astrocytes into neurotoxic A1 phenotype. M1 microglia and A1 astrocytes release pro-inflammatory cytokines, which increase BBB permeability, promoting the infiltration of peripheral T cells and monocytes across the BBB and aggravating the neurodegeneration of dopaminergic neurons. BBB, blood-brain barrier; α-syn, alpha synuclein; TNF-α, tumor necrosis factor alpha; IL-1β, interleukin-1β; C1q, complement component 1q; ROS, reactive oxygen species; LPS, lipopolysaccharide; TGF-β, transforming growth factor beta; IGF, insulin-like growth factor; IFN‐γ, interferon gamma

Numerous PD models have demonstrated notable microglial activation in response to various toxins, including rotenone, 1-methyl-4-phenyl-1,2,3,6-tetrahydropyridine (MPTP), and 6hydroxydopamine hydrochloride (6-OHDA), as well as α-syn transgenic animal models [37]. When microglia undergo activation, their resting, ramified phenotype has transformed into an intermediate, hyper-ramified morphology, characterized by a large soma and an amoeboid shape, becoming more mobile to start phagocytosis (Fig. 3). Microglia can exhibit either an M1 phenotype linked to neurotoxicity or an M2 phenotype linked to neuroprotection when they are activated [38]. While chronic activation of microglia has been suggested as a possible mechanism in neurodegenerative disorders, short-term (acute) activation of microglia has been widely considered neuroprotective [39].

The M1 phenotype microglia have been activated by interferon-gamma (IFN-γ) and lipopolysaccharide (LPS), producing high levels of pro-inflammatory signaling molecules such as TNF-α, IL-1β, and IL-12, which have been associated with dopaminergic neuron degeneration in PD [38, 40]. Conversely, the M2 microglia have been activated by IL-4, IL-10, or IL-13, releasing a variety of anti-inflammatory cytokines, including transforming growth factor beta (TGF-β), insulin-like growth factor-1 (IGF-1), IL-4, IL-13, and IL-10, to reduce inflammation and enhance the healing process [41].

Astrocytes are the most prevalent glial cells in the CNS [42]. Their numerous extensions were found to be directly connected to neurons and blood vessels in the blood-brain barrier (BBB) through gap junctions, forming the neurovascular unit. Thus, astrocytes are important modulators of neuronal activity and cerebral blood flow and contribute to the permeability and maintenance of the BBB [43]. Astrocytes have been reported to have particular shuttle systems, including glutamate–glutamine and malate–aspartate fluxes, providing lactate for mitochondrial respiration, which metabolically can support neurons [44]. Additionally, astrocytes may contribute to tissue repair by occupying the spaces left by dead neurons and releasing trophic factors necessary for synaptic function and neuron survival [45].

Astrocytes have been categorized into A1 neurotoxic astrocytes and A2 neuroprotective astrocytes (Fig. 3). Activated microglia can secrete complement component 1q (C1q), IL-1, and TNF-α, which can activate astrocytes toward an A1 neurotoxic phenotype, thereby releasing pro-inflammatory cytokines including, TNF-α, IL-1β, IFN-γ, IL-1, and IL-6, as well as inflammatory chemokines, including CCL2, CXCL1, and CXCL2. These molecules may promote the recruitment of circulating immune cells to enter the lesion site and enhance the activation and aggregation of inflammatory cells [46, 47]. The activation of astrocytes toward an A1 phenotype has been reported to depend on the presence of microglia and sufficient levels of IL-1α, TNF, and C1q, as the administration of LPS to animals lacking microglia does not induce astrocyte activation [48].

Alternatively, A2 reactive astrocytes have been considered neuroprotective because they have increased the production of neurotrophic factors in response to insults, such as ischemia. A2 astrocytes can release anti-inflammatory substance such as TGF-β and IL-10, mediate tissue repair, protect against oxidative stress, preserve neuronal homeostasis, and prevent reactive microglial accumulation [47]. Eight of the 17 monogenic genes that are known to play a part in the development of PD are expressed in astrocytes at the same level as or higher than those in neurons; these are PARK7, SNCA, PLA2G6, ATP13A2, LRRK2, GBA, PINK1, and PARK2. A few of these genes have a strong connection to inflammation [49].

### Mitochondrial Dysfunction Cross-link with Neuroinflammation in Parkinson’s Disease

Growing evidence has demonstrated a complex and dynamic bidirectional relationship between neuroinflammation and mitochondrial dysfunction. After exposure of neurons or glia to external stimuli, mitochondrial function becomes impaired, and the mitochondrial membrane is compromised, resulting in the release of mitochondrial ligands into the cytoplasm or outside the cell. These ligands, referred to as mitochondrial-DAMP, exacerbate neuroinflammation by binding to pattern recognition receptors on microglia [50].

Conversely, activated glial cells release pro-inflammatory cytokines that initiate an intracellular cascade controlling mitochondrial metabolism and function, thereby creating an endless cycle of neuroinflammation and mitochondrial dysfunction [51]. After significant mitochondrial damage, mitochondrial DNA may evade mitophagy and activate IFN-related genes, TLR9, and the NOD-like receptor protein 3 (NLRP3) inflammasome. Cytosolic mitochondrial DNA can function as a DAMP to regulate the inflammatory response. NLRP3 activation promotes caspase-1 activation and the maturation and release of IL-18 and IL-1β, thereby intensifying the inflammatory response [52].

Mitochondrial dysfunction is exacerbated by mutations in mitochondrial DNA, increased ROS production, alterations in mitochondrial dynamics, and the loss of mitochondrial membrane potential. Neurons are highly sensitive to mitochondrial dysfunction due to their high energetic demands. Remarkably, the mitochondrial quality control pathway has been closely linked to several PD-related genes, including PINK1 and Parkin [53]. The mitochondrial electron transport chain and mitochondrial NADPH oxidase have been considered the major mitochondrial ROS sources. Excessive mitochondrial ROS production can destroy mitochondrial components in addition to destroying proteins and lipids, thereby aggravating mitochondrial dysfunction [54, 55].

Mitochondrial dysfunction in glial cells has been associated with excessive mitochondrial ROS production and the release of cardiolipin from the inner mitochondrial membrane into the cytoplasm. Evidence has strongly supported the role of mitochondrial ROS and cardiolipin in NLRP3 inflammasome activation. Under basal conditions, the NLRP3 inflammasome has been reported to localize in the endoplasmic reticulum. In response to ROS production, NLRP3 redistributes close to the nucleus, binds to the mitochondria, and assembles into an active inflammasome complex [56].

**Fig. 4:**
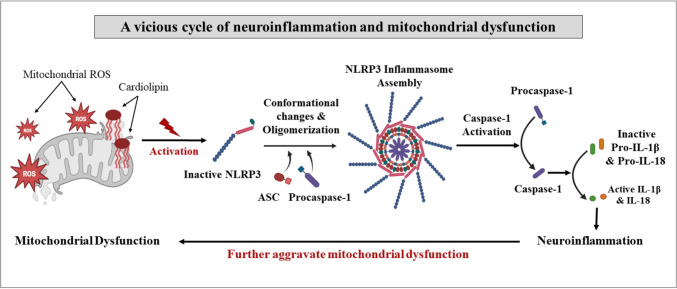
Schematic presentation of the bidirectional relationship between neuroinflammation and mitochondrial dysfunction in Parkinson’s disease. Mitochondrial dysfunction in neurons or glia cause the release of certain molecules, like cardiolipin and ROS. Those molecules trigger the NLRP3 conformational changes and oligomerization with ASC adaptor and procaspase-1 to form the active NLRP3 inflammasome complex, thereby activating procaspase-1 into caspase-1. Caspase-1 triggers the activation of pro-IL-1β and pro-IL-18 into their mature forms (IL-1β and IL-18), subsequently causing neuroinflammation. In turn, neuroinflammatory mediators may further aggravate mitochondrial dysfunction and ROS production, creating a self-perpetuating cycle of neuroinflammation and mitochondrial impairment. ROS, reactive oxygen species; ASC, apoptosis-associated speck-like protein containing a caspase recruitment domain; NLRP3, the NOD-like receptor protein 3; IL, interleukin

In response to cellular stress signals, the NLRP3 protein undergoes conformational changes and oligomerizes into a multimeric complex (Fig. 4). This oligomerization promotes recruitment of the adaptor protein ASC and procaspase-1, leading to cleavage procaspase-1 into active caspase-1. Caspase-1 is then able to convert the pro-inflammatory cytokines IL-1β and IL-18 into their mature forms, thereby inducing inflammation [57].

## Biogenesis of CircRNAs

In contrast to canonical splicing, circRNAs, members of the non-coding RNA (ncRNA) family, are generated from pre-mRNA (precursor messenger RNA) by back splicing or exon skipping to form covalently closed loop structures without 5′ to 3′ ends. In back splicing, a downstream 5′ splice donor is joined to an upstream 3′ splice acceptor, resulting in circRNA formation. They are stable in the cytoplasm and accumulate intracellularly due to their lack of free ends, which makes exosomal excretion a crucial removal mechanism [58]. The majority of circRNAs are produced from pre-mRNAs, but some are made from pre-tRNAs (precursor transfer RNA) or other RNA species [11].

**Fig. 5:**
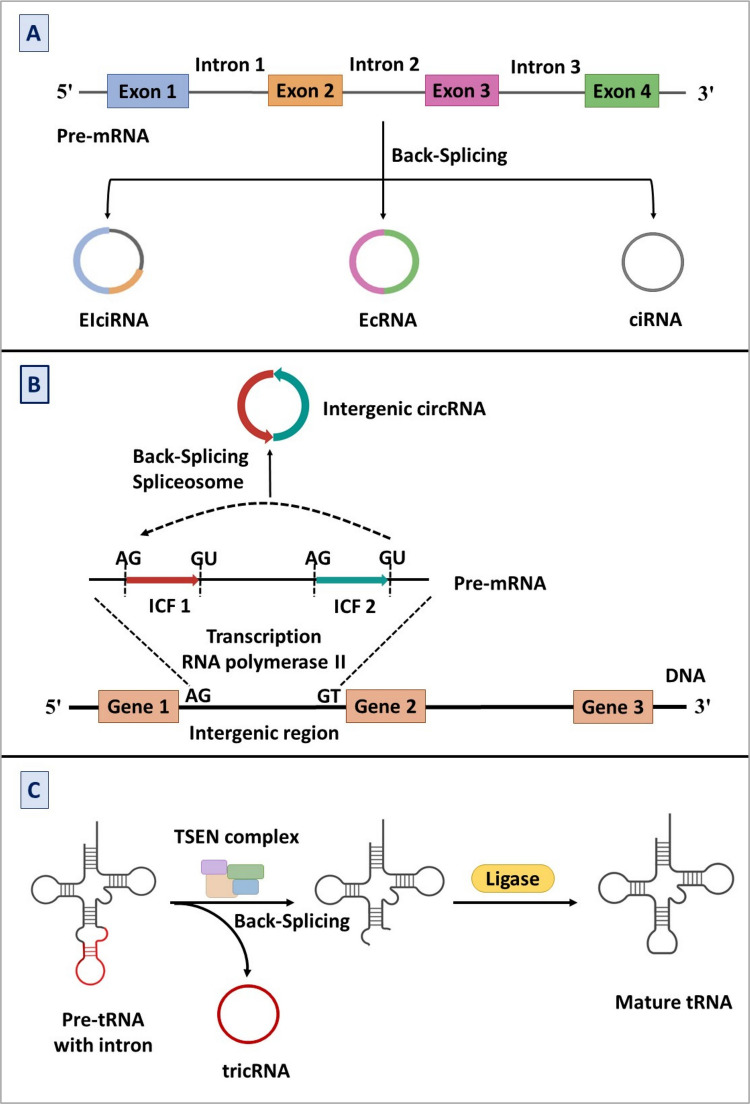
Biogenesis of different types of circRNA. **A** Exonic circRNAs (EcRNAs), which originate from exons only; intronic circRNAs (ciRNAs), which only contain introns; and exon-intron circRNAs (EIciRNAs), which are circularized with both exons and introns. **B** Intergenic circRNA. **C** tRNA intronic circular RNAs (tricRNAs). pre-mRNA, precursor messenger RNA; pre-tRNA, precursor transfer RNA; TSEN, tRNA splicing endonuclease

Exons, introns, intergenic regions, antisense RNAs, and/or untranslated regions (UTRs) can all produce these RNA molecules. As shown in Fig. 5, circRNAs can be divided according to their origin into exonic circRNAs (EcRNAs), which are derived from exons; intronic circRNAs (ciRNAs), which consist only of introns; exon-intron circRNAs (EIciRNAs), which contain both exons and introns; intergenic circRNAs; and tRNA intronic circular RNAs (tricRNAs). The majority of EcRNAs have been exported to the cytoplasm, while ciRNAs and EIciRNAs have been found in the nucleus. Among the circRNAs found, EcRNAs make up more than 80% [59, 60].

Intergenic circRNAs are a non-exonic class of circRNAs produced from intergenic regions of the genome through back-splicing of pre-mRNA (Fig. 5). Intergenic circRNAs have been reported to be generated from two intronic circRNA fragments (ICF), accompanied by GT-AG splicing motifs [61]. In eukaryotic pre-mRNA, the spliceosome recognizes these motifs at the ends of introns during RNA splicing [62]. The tricRNAs are a specific type of circRNA created following pre-tRNA splicing by excising and circularizing tRNA introns (Fig. 5). The hetero-tetrameric tRNA splicing endonuclease (TSEN) complex, composed of four proteins, mediates pre-tRNA splicing in eukaryotes. Following cleavage by TSEN, certain RNA ligases ligate the tRNA halves to create a mature tRNA, and they also ligate the intron termini to create a tricRNA [63, 64].

## Mechanisms of Action of CircRNA

CircRNAs can be participate in wide range of biological processes; they can influence nearly every stage of a gene’s life cycle [65]. Currently, the most extensively investigated and widely acknowledged mechanism regarding the regulatory role of circRNAs on gene expression is the competing endogenous RNA (ceRNA) hypothesis. CircRNAs contain many miRNAs binding sites, known as miRNA responsive elements (MREs), and act as miRNA sponges to sequester miRNAs. This binding between circRNAs and miRNAs prevents miRNA from binding with the 3′-untranslated region of mRNA, which either inhibits translation or promotes mRNA degradation [66]. The most typical miRNA sponge is CDR1as. It has been reported that this circRNA have more than 70 conserved binding sites for miR-7, which significantly lowers the amount of miR-7 when it upregulated [67].

CircRNAs can also function as protein sponges through binding with RNA-binding proteins (RBPs), a large class of proteins that are involved in the transcription and translation of genes. This is a crucial function of circRNA, which can affect RNA splicing, transport, and degradation by controlling the location, activity, or stability of RBPs [68]. For instance, circMb1 can participate in gene regulation by competing with linear splicing through a strong direct contact with the MBL protein [69]. CircPABPN1 can inhibit PABPN1 translation by competitively binding to HuR and blocking HuR’s interaction with PABPN1 mRNA [70].

Although circRNAs are classified as ncRNAs, it is more interesting that certain circRNAs can be translated into proteins or functional peptides that have regulatory functions. These circRNA molecules contain key translational features such as open reading frames, N6-methyladenosine, and the internal ribosome entry site, which are among the crucial components required for the translation initiation step [71]. Experimental studies have shown that some circRNAs can be translated into proteins, including circRNA-derived proteins identified in human cancer such as circ-FBXW7 and a tumor-suppressive protein encoded by circ-SHPRH [72, 73 ]. These findings indicate that circRNAs with coding potential are widely distributed, providing a new possibility for the functional investigation of circRNAs.

Additionally, it has been documented that nuclear circRNAs, such as ciRNAs and EIciRNAs, mediate the transcriptional and posttranscriptional expression of parental genes. At the transcriptional level, EIciRNAs, such as circEIF3J and circPAIP2, can interact with U1 small nuclear ribonucleic proteins and form a complex with RNA polymerase II to upregulate the expression of parental genes. They can also control the posttranscriptional mechanisms, such as selective splicing [65, 74].

## CircRNAs as Biomarkers in Parkinson’s Disease

Evidence indicates that circRNAs have several of properties over linear RNA, including high stability, broad distribution, tissue-specificity, developmental stage- or age-dependent expression, and evolutionary conservation across different species, making them promising biomarker for diseases. The unique covalently closed-loop structure of circRNAs, which lacks free 5′ and 3′ ends, contributes to their stability of circRNAs and long half-life. Most exonic circRNAs have a half-life longer than 48 h, whereas mRNAs have a half-life around 10 h [75]. Compared with mRNAs, both exonic circRNAs and intronic circRNAs have been considered to be more stable and resist ribonuclease R (RNase R) digestion [76, 77 ]. CircRNAs have been thought to be more abundant in the brain than other organs, and because of their highly stable structure, circRNAs are also widely distributed in the blood [76, 78]

In addition, many circRNAs show tissue-specific expression patterns and vary with developmental stage or age, further increasing their potential as biomarkers for specific diseases. For example, circRNAs derived from RMST and KLHL2 are highly expressed in the mouse brain but not in the liver or lung, highlighting the tissue-specific enrichment of circRNAs in the CNS [79]. Furthermore, evidence demonstrated that the expression of many circRNAs, including mm9_circ_013636 and mm9_circ_004501, was elevated in the brain of 22-month-old mice compared with 1-year-old mice [80]. In addition, comparative analyses have identified 457 human circRNAs that map to murine genes, of which 69 exhibit conserved back-splicing junctions, suggesting that a group of circRNAs is evolutionarily conserved across species. This indicates that certain circRNA biomarkers found in murine models may have clinical applications in humans [81].

CircRNAs have been found in high amount in body fluids, including blood, CSF, saliva, and urine. Additionally, they can be particularly identified in circulating blood cells and tumor cells [82]. Although circRNAs are detectable in peripheral biofluids and CSF, current evidence does not yet establish that they freely cross the BBB as intact molecules. Many circRNAs are enriched in extracellular vesicles (EVs), including EVs found in plasma and CSF, suggesting that vesicle-mediated transport and/or BBB disruption may contribute to their appearance in biofluids [83].

In a study of peripheral blood exosomes from PD patients, researchers found many circRNAs were differentially expressed compared with healthy controls, and some changed further after rehabilitation. Two circRNAs, has_circ_0001535 and has_circ_000437, were selected for validation and were both reduced after rehabilitation. Network analysis suggested that has_circ_0001535 may be related to α-syn aggregation and has_circ_000437 may regulate inflammatory responses. These findings suggest that circRNAs in peripheral exosomes may serve as potential biomarker for PD and rehabilitation response [84]. Several circRNAs, including MAPK9_circ_0001566, SLAIN1_circ_0000497, SLAIN2_circ_0126525, PSEN1_circ_0003848, circ_0004382, and circ_0017204, have been proposed as circulating biomarkers for PD [85]. However, human studies remain limited, and larger independent cohorts are needed to validate the diagnostic and translational value of circRNAs as clinically useful biomarkers.

## The Role of Circular RNAs in Parkinson’s Disease-Related Neuroinflammation

**Fig. 6:**
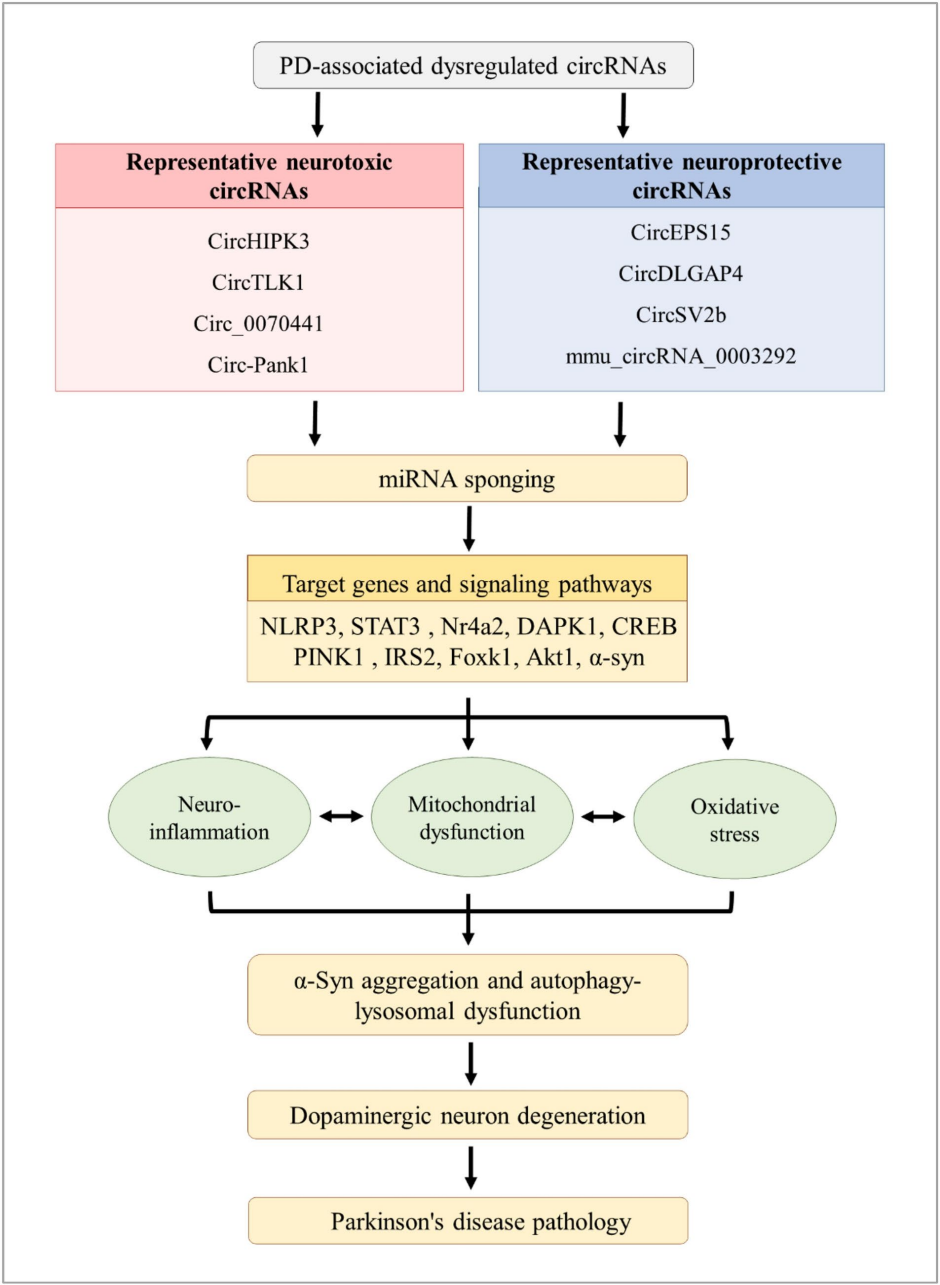
Linking circRNAs, inflammation, mitochondrial dysfunction, and oxidative stress in Parkinson’s disease. Representative circRNAs in Parkinson’s disease function as competing endogenous RNAs by sponging miRNAs and thereby modulating downstream target genes and signaling pathways. These circRNA-mediated interactions converge on major pathological process, including neuroinflammation, mitochondrial dysfunction, oxidative stress, impaired autophagy-lysosomal clearance, α-syn aggregation, and dopaminergic neuron degeneration. Collectively, these pathways contribute to Parkinson’s disease progression and may also provide potential diagnostic and therapeutic targets. CircHIPK3, CircRNA homeodomain-interacting protein kinase 3; STAT, signal transducer and activator of transcription; Nr4a2, nuclear receptor 4a2; DAPK1, death-associated protein kinase 1; CREB, cAMP-response element binding protein; IRS2, insulin receptor substrate 2

Research has indicated that circRNAs can regulate the immune and inflammatory pathways and alter mitochondrial function, influencing ROS generation within mitochondria, which in turn intensifies inflammatory signaling in neurodegenerative disorders. Specifically, circRNAs can influence inflammation in PD by serving as miRNA sponges and modifying important signaling pathways, highlighting the dual role of circRNAs in mediating both neuroprotective and neurotoxic effects in PD (Fig. 6). Table 1 summarizes circRNAs that proved neuroprotective and neurotoxic action in PD and related neuroinflammatory disorders.

**Table 1:** Circular RNAs having neuroprotective/neurotoxic effects in Parkinson’s disease and related neuroinflammatory disorders

	circRNA	Target miRNA/gene	Effects	Source of evidence	Ref
**PD specific models**	CircHIPK3	miR-124/STAT3 and NLRP3	CircHIPK3 has a neurotoxic effect through enhancing the inflammatory response and increasing ROS production in PD patients and LPS-induced BV2 cells	Human study and cell model	[86]
	mmu_circRNA_0003292	miR-132/Nr4a2	mmu_circRNA_0003292 has a neuroprotective effect through regulating the differentiation of dopaminergic neurons, immune responses, and neuronal survival in the PD animal model	Animal model	[87]
	CircTLK1	miR-26a-5p/DAPK1	CircTLK1 has a neurotoxic effect by increasing the expression of DAPK1, which may exacerbate neuronal damage, and influences inflammation and the innate immunity response in the CNS in the PD model	Animal model and cell model	[88]
	CircDLGAP4	miR-134-5p/CREB	CircDLGAP4 has a neuroprotective effect through enhancing neuronal viability, reducing apoptosis, and reducing mitochondrial damage in PD	Animal model and cell model	[89]
	CircEPS15	miR24-3p/PINK1	CircEPS15 has a neuroprotective effect that protects dopaminergic neurons against neurotoxin-induced PD and improves mitochondrial function	Human study, animal model, and cell model	[90]
	CircSV2b	miR-5107-5p/Foxk1/Akt1	CircSV2b has a neuroprotective effect by protecting dopaminergic neurons from oxidative damage and promoting neuronal survival	Animal model and cell model	[91]
	Circ_0070441	miR-626/IRS2	Circ_0070441 has a neurotoxic effect by stimulating apoptosis, inflammation, and neuronal damage in an in vitro cellular PD model	Cell model	[92]
	Circ-Pank1	miR-7a-5p/α-syn	Circ-Pank1 has a neurotoxic effect by increasing the production of α-syn and promoting locomotor dysfunction in the PD mouse model	Animal model and cell model	[16]
	CircSLC8A1	miR-128/BMI1, AXIN1, and SIRT1	CircSLC8A1 has a neurotoxic effect by dysregulating genes linked to aging and neurodegeneration and contributing to PD progression	Human study and cell model	[93]
	Hsa_circ_0001535 (circFAM13B)	miR-212/P53	Hsa_circ_0001535 has a neurotoxic effect by causing autophagic damage to dopaminergic neurons, aberrant α-syn aggregation, and ROS production in the PD model	Human study	[84]
	Hsa_circ_0004381	miR-185-5p/RAC1	Hsa_circ_0004381 has a neurotoxic effect by driving oxidative stress, inflammatory response, and apoptosis, while reducing cell survival	Cell model	[96]
**Related neuroinflammatory disorders**	Circ-001372	PIK3CA/Akt/NF-κB signaling pathway	Circ-001372 has a neuroprotective effect by reducing inflammation and neural apoptosis in a propofol-induced neurotoxicity and neuroinflammation	Animal model and cell model	[97]
	Circ-0000220	miR-326-3p/BCL3, MK2, TRIM25	Circ-0000220 has a neurotoxic effect that indirectly induces the production of inflammatory cytokines, thereby positively regulating the JEV-induced inflammatory response in BV-2 microglial cells	Animal model and cell model	[99]
	CircRNA SCMH1	STAT5B/KMO	CircRNA SCMH1 has a neuroprotective effect by reducing astrocytic and microglial reactivity, inhibiting mitophagy, and promoting mitochondrial fusion after cerebral ischemia	Animal model	[101]
	CircDYM	miR-9/HECTD1	CircDYM has a neuroprotective effect by reducing pro-inflammatory cytokines and inhibiting microglial activation through increasing HSP90 ubiquitination in MDD	Human study, animal model, and cell model	[104]
	CircPTP4A2	STAT3	CircPTP4A2 has a neurotoxic effect by enhancing microglia M1 polarization and neuroinflammation after ischemic stroke through activation of STAT3	Animal study and cell model	[105]
	CircPRKCI	miR-545,miR-589/E2F7	CircPRKCI has a neuroprotective effect by protecting neurons from oxidative damage	Cell model	[106]

Brain fluids and serum from PD patients, as well as LPS-induced BV2 cells, showed elevated *circHIPK3* (circRNA homeodomain-interacting protein kinase 3) expression, while *miR-124* expression was significantly downregulated. Prior research has also demonstrated that *miR-124* plays a regulatory role in neuroinflammation, autophagy, mitochondrial dysfunction, oxidative damage, and cell survival in PD. *CircHIPK3* may act as a ceRNA by sponging *miR-124*, thereby enhancing the inflammatory response through the activation of the NLRP3 and signal transducer and activator of transcription-3 (STAT3) signaling pathways. In LPS-stimulated BV2 cells, *circHIPK3* overexpression increased ROS production, and this effect was mitigated by *miR-124*. Conversely, *circHIPK3* silencing reduced LPS-induced oxidative damage and inflammation, suggesting that *circHIPK3* may be a viable target for PD treatment [86].

Research also demonstrates that *mmu_circRNA_0003292* controlled nuclear receptor 4a2 (Nr4a2) expression by sponging *miR-132* in MPTP-induced animal models of PD. The overexpression of *miR-132* decreased dopamine neuron differentiation by directly targeting Nr4a2. Nr4a2, a transcription factor for the development and differentiation of dopamine neurons in the brain, has been suggested to be essential for developing and maintaining the activities of mesencephalic dopaminergic neurons. Therefore, through modulation of Nr4a2, the *mmu-circRNA-0003292/miRNA-132* axis has been linked to immunological modulation and neuronal survival. These outcomes may indicate that circRNAs have a role in controlling immunological responses in the brains of PD patients [87].

Chen et al*.* demonstrated that *circTLK1* can act as a sponge for *miR-26a-5p*, thereby raising the expression of death-associated protein kinase 1 (DAPK1), which may exacerbate neuronal damage. Additionally, it has been suggested that DAPK1 activation may influence inflammation and the innate immunity response in the CNS in both in vitro and in vivo PD models through its involvement in the production of IL-1β and caspase-1 activation. Conversely, circTLK1 deficiency decreases dopaminergic neuron damage both in vitro and in vivo by releasing *miR-26a-5p*, which targets DAPK1. Consequently, targeting *circTLK1* may help improve PD treatment [88].

Furthermore, *circDLGAP4* has been found to work as a *miR-134-5p* sponge to shield cAMP response element binding protein (CREB) from the suppression effect caused by *miR-134-5p*. Activation of CREB can promote the transcription of downstream target genes, including brain-derived neurotrophic factor, B-cell lymphoma 2, and peroxisome proliferator-activated receptor gamma coactivator-1α, resulting in the protective effect on the brain. Moreover, the overexpression of *circDLGAP4* enhances neuronal viability, lowers apoptosis, and lowers mitochondrial damage in PD [89].

The *circEPS15* overexpression has been found to protect the dopaminergic neurons from neurotoxin-induced PD in vitro and in vivo through improving mitochondrial function via the *miR-243p*/PINK1 axis. *CircEPS15* has been found to act as a *miR-24-3p* sponge, thereby improving PINK1-PRKN-dependent mitophagy, which removes damaged mitochondria and preserves mitochondrial homeostasis. Moreover, it was found that there is a correlation between the severity of PD motor symptoms and the *circEPS15* level in PD patients. Thus, *circEPS15* could play a crucial role in the pathophysiology of PD and could provide new insights for the development of PD biomarkers and treatment targets [90].

In PD models, *CircSV2b* was found to be downregulated. Overexpression of *circSV2b* decreased oxidative stress damage by sponging *miR-5107-5p*, which subsequently led to the upregulation of Foxk1 and the activation of Akt1 signaling. This regulatory axis could protect dopaminergic neurons from oxidative damage and promote neuronal survival. Furthermore, the study emphasized a significant alteration of *circSV2b* in serum exosomes and suggested that *circSV2b* may be a new biomarker for PD diagnosis and therapy [91].

Cao et al*.* studied the *circ_0070441/miR-626*/insulin receptor substrate 2 (IRS2) regulatory axis in MPP^+^-treated SH-SY5Y cells as an in vitro cellular PD model. MPP+ induction may significantly increase the secretion of pro-inflammatory cytokines (TNF-α, IL-1β, and IL-6). Knockdown of *circ_0070441* partially reduced the inflammation and neuronal apoptosis in SH-SY5Y cells. *Circ_0070441* can act as a sponge for *miR-626*, thereby increasing IRS2 expression, which in turn stimulates apoptosis, inflammation, and neuronal damage. These results may suggest a new regulation mechanism in PD mediated by ceRNAs, which could lead to a new therapeutic target [92].

Liu et al*.* found that *circ-Pank1* exhibits elevated levels in both the SN of PD mice model and the MN9D cell model of dopaminergic neurons. *Circ-Pank1* has been shown to sponge *miR-7a-5p* and increase the production of α-syn, a biological marker that is strongly associated with PD. Blocking of circPank1 alleviated dopaminergic neuronal injury and motor dysfunction, while the suppression of *miR-7a-5p* reversed this effect. In summary, *circ-Pank1* has been suggested to be upregulated in PD and promote locomotor dysfunction through the *miR-7a-5p*/α-syn regulatory axis, demonstrating a possible therapeutic target to slow disease progression [16].

It has been reported that *circSLC8A1* is markedly overexpressed in the SN of PD patients and strongly correlates with oxidative stress and other PD-inducing factors. After additional analyses to validate the relationship between *circSLC8A1* and *miR-128* in PD, it was found that *circSLC8A1* could regulate *miR-128* activity without degrading it, thereby indirectly deregulating *miR-128* targets associated with PD, including the neurodegeneration- and aging-related B lymphoma Mo-MLV insertion region 1 homolog (BMI1), axis inhibition protein 1 (AXIN1), and Sirtuin 1 (SIRT1). Consequently, the *circSLC8A1/miR-128* axis may also be viewed as a regulatory mechanism during the progression of PD [93].

Validation results showed that PD patients had significantly higher expression levels of *hsa_circ_0001535* (*circFAM13B*) than their levels in normal controls. *CircFAM13B* has a sponging effect on *miR-212* and may activate the p53 pathway [84]. Recent studies in PD patients and cellular and animal models have shown that p53 activity and levels are significantly higher in damaged neurons. Autophagic damage to dopaminergic neurons, aberrant protein aggregation, and ROS production are all closely linked to p53 activation in response to neurodegenerative stress, and these pathogenic events are related to the pathophysiology of PD [94]. Other researchers have discovered that α-syn, promoter activity, and mRNA levels are significantly increased when p53 expression is upregulated genetically and pharmacologically [95].

Zhang et al*.* studied the role of the *hsa_circ_0004381/miR-185-5p*/Rac family small GTPase 1 (RAC1) in 1-methyl-4-phenylpyridinium (MPP^+^)-induced SK-N-SH cells as an in vitro model of PD. According to mechanical analyses, *hsa_circ_0004381* can influence RAC1 expression by acting as a sponge for *miR-185-5p*. This study demonstrated that the expression of *miR-185-5p* decreased, whereas the expression of *hsa_circ_0004381* and RAC1 increased in the PD cell model. Moreover, silencing *hsa_circ_0004381* increased cell survival while suppressing oxidative stress, inflammatory response, and apoptosis. Thus, *hsa_circ_0004381* may target the *miR-185-5p*/RAC1 axis and contribute to neuronal damage brought on by MPP+, offering new perspectives on the causes and therapies of PD [96].

In a rat model of neurotoxicity and neuroinflammation caused by propofol anesthesia, *circRNA001372* can inhibit neurotoxicity and neuroinflammation via the PI3K (phosphoinositide 3kinase)/Akt/NF-κB signaling pathway. Inhibition of *circRNA001372* in propofol-treated cells may cause inflammation and neural apoptosis by raising cytokines, such as IL-1β, IL-6, IL-17, and IL-18, leading to a reduction in cell viability. The overexpression of *circRNA001372* counteracted these effects and provided a neuroprotective effect. It was discovered that the *miRNA-148b-3p* is a neurotoxic miRNA that increases NF-κB level, which was decreased by *circRNA001372*, while decreasing the PI3K and pAKt levels, which were increased by *circRNA001372* [97].

The neuropathogenesis of the Japanese encephalitis virus (JEV) has been defined by the trigger of a strong inflammatory response and the subsequent death of neurons. The main cause of JEV-induced neuroinflammation has been suggested to be the unregulated activation of microglia, which release a lot of inflammatory cytokines and chemokines [98]. Silencing of *circ_0000220* in BV2 cells before JEV infection greatly decreased the production of inflammatory cytokines. Similarly, overexpression of *miR-326-3p* resulted in a marked decrease in inflammatory cytokine expression. According to a previous study, *circ_0000220* sponges the *miR-326-3p* and thereby influences the expression of its target genes, including B-cell leukemia/lymphoma 3 (BCL3), mitogen-activated protein kinase-activated protein kinase 2 (MK2), and tripartite motif-containing protein 25 (TRIM25), positively regulating the JEV-induced inflammatory response [99].

Following cerebral ischemia, *circSCMH1* may dramatically lower astrocytic and microglial reactivity [100]. Wang et al*.* demonstrated that *circSCMH1* could inhibit mitophagy and promote mitochondrial fusion by directly binding to STAT5B and reducing its nuclear translocation, which consequently decreases the expression of kynurenine 3-monooxygenase (KMO), as STAT5B binds to the promoter of the KMO gene to promote its expression [101]. KMO has been considered a key player in metabolic regulation and is primarily expressed in microglia. Pro-inflammatory cytokines increase KMO substrate and expression levels, while functional genetic mutations change these levels. Whereas research has shown that decreased KMO activity can have neurodevelopmental effects, increased KMO metabolism produces metabolites that increase oxidative stress and activate glutamate receptors [102, 103].

According to a previous study, *circDYM* could be a new therapeutic target for major depressive disorder (MDD). It was discovered that *circDYM* was downregulated in the hippocampus and plasma of mouse models that resembled depression, as well as in the peripheral blood of MDD patients. *CircDYM* acts as a *miR-9* sponge, controlling downstream *miR-9* target genes, such as HECT domain E3 ubiquitin protein ligase (HECTD1). As a result of reduced *circDYM* expression, HECTD1 has been downregulated, increasing the bioavailability of *miR-9*. Thus, reduced HECTD1-mediated HSP90 ubiquitination increases the pro-inflammatory cytokines and induces neuroinflammation and microglial activation [104].

A mouse model of transient middle cerebral artery occlusion showed markedly elevated levels of *circPTP4A2* in both plasma and the peri-infarct cortex. Knockdown of *circPTP4A2* attenuated neurological deficits, increased cortical cerebral blood flow, and decreased infarct volume. Additionally, 24 h after the stroke, knockdown of *circPTP4A2* facilitated the phenotypic change of microglia from an M1 to an M2 state, which may be caused by the downregulation of phosphorylated STAT3, indicating possible immunoregulatory mechanisms of *circPTP4A2* following ischemic stroke. According to these findings, *circPTP4A2* regulatory mechanisms may reduce neuroinflammation by controlling microglial polarization and provide theoretical support for its possible use as a therapeutic target in ischemic stroke treatment [105].

Furthermore, the overexpression of *circPRKCI* significantly diminished H₂O₂-induced neuronal cell damage, by sponging *miR-545/589*, and upregulated the expression of its target, E2F7. According to these findings, H₂O₂-induced neuronal cell damage was due to dysregulation of the *circPRKCI*-*miR-545/589*-E2F7 axis. Focusing on this new signaling pathway may be a promising strategy to shield neurons from oxidative damage as well [106].

## Future Perspectives

CircRNAs, miRNAs, and their target genes have been found to form complex regulatory axes that are crucial in the pathophysiology of PD and other neurodegenerative diseases, according to extensive research. Despite the experimental validation of individual interactions (such as circRNA/miRNA or miRNA/mRNA), there are still few comprehensive studies that address the entire triad (circRNA/miRNA/mRNA axis) in a single experimental framework. This represents a significant knowledge gap, as these triads may serve as master regulators of neuroinflammation in neurodegenerative disorders.

For instance, recent research has indicated that *circHIVEP2* targets *miR-485-3p* to reduce inflammation and neuronal damage in PD. The results showed that while *miR-485-3p* was upregulated in PD, *circHIVEP2* was significantly downregulated. Another significant study on *miR-485-3p* and FBXO45 in PD confirmed that FBXO45 may be a *miR-485-3p* target, indicating that *miR-485-3p* regulates neuroinflammatory responses in PD and may serve as a therapeutic target and diagnostic biomarker. Serum *miR-485-3p* expression was higher in patients with PD than in those with AD or healthy controls. Reducing the expression of *miR-485-3p* limited the release of inflammatory cytokines from microglia cells, thereby regulating neuroinflammation [107]. Since *circHIVEP2* has been reported to regulate *miR-485-3p*, which independently targets FBXO45, it can be hypothesized that a regulatory axis involving *circHIVEP2*/*miR-485-3p*/FBXO45 exists in PD, contributing to the modulation of neuroinflammation and neurodegeneration processes.

Another example demonstrated that *circSLC8A1* was upregulated in the substantia nigra tissue of PD patients and contains several *miR-128* binding sites. In cell culture models, silencing of *circSLC8A1* altered the expression of *miR-128* target genes, indicating that *circSLC8A1* functions as a *miR-128* sponge and influences its regulatory activity [108]. In PD, another study found that *miR-128* has an essential neuroprotective role in the oxidative stress response and neuronal death. Circulating exosomes from PD patients and cellular PD models showed a significant reduction in *miR-128*. Overexpression of *miR-128* prevents neuronal death brought on by toxins like 6-OHDA by downregulating the transcription factor FOXO3a (forkhead homeobox type O) and its pro-apoptotic substrates FasL (Fas ligand) and PUMA (p53 upregulated modulator of apoptosis), which in turn suppresses the activation of caspases-8, −9, and −3 [109]. Although the connection between *circSLC8A1* binding *miR-128* and *miR-128* controlling FOXO3a has been suggested, it has not been experimentally verified as a complete pathway in a single PD-focused investigation.

Understanding the circRNA/miRNA/mRNA network may facilitate biomarker discovery by identifying coordinated regulatory signatures linked to neuroinflammation. For example, upregulation of a specific circRNA may decrease the availability of its target miRNA, thereby increasing downstream inflammation-related mRNAs. This reproducible three-layer pattern would provide stronger biomarker evidence than an isolated RNA change because it reflects direction, mechanistic relevance, and a direct connection to PD-associated inflammatory pathways. CircRNAs and miRNAs are particularly attractive biomarker candidates because of their relative stability and presence in accessible biofluids such as blood, plasma, serum, exosomes, and cerebrospinal fluids. Moreover, disease-associated circRNA/miRNA/mRNA networks may help identify molecular signatures associated with disease stage and progression. To translate these findings into clinically relevant applications, future studies should combine multi-omics approaches with in vivo validation and well-characterized clinical cohorts.

## Conclusion

Recent evidence indicates that circRNAs play an important role in the pathophysiology of PD by regulating neuroinflammation, immune response, mitochondrial dysfunction, ROS accumulation, and neuronal survival through circRNA/miRNA/mRNA regulatory networks. In particular, several circRNAs have been shown to modulate key PD-related signaling pathways, including NLRP3, STAT3, NF-kB, α-syn aggregation, and PINK1-mediated mitophagy, suggesting that they participate in multiple interconnected mechanisms that contribute to dopaminergic neuron degeneration. These findings support the view that circRNAs are not only involved in disease progression but may also serve as promising biomarkers and therapeutic targets for PD. Their high stability, tissue-specific expression, and detectability in biofluids such as blood and CSF make them especially attractive candidates for non-invasive diagnosis and disease monitoring.

Despite these encouraging findings, most current studies are based on in vitro experiments and in animal models, and relatively few have been validated in human samples. In addition, longitudinal clinical cohorts are still lacking, and the mechanistic relationships between individual circRNAs, their target miRNAs, and downstream effector pathways remain incompletely understood in vivo. Therefore, further studies are needed to determine whether circRNAs can reliably reflect disease onset, progression, and treatment response in patients with PD. Future research should focus on large-scale CSF and plasma profiling, multi-omics integration, and functional validation of pathogenic circRNAs to establish their diagnostic and prognostic value. In parallel, therapeutic strategies targeting disease-associated circRNAs may provide new avenues for intervention in PD, particularly through modulation of inflammatory and mitochondrial pathways that drive dopaminergic neurodegeneration.

## Data Availability

No datasets were generated or analysed during the current study.
